# Comparative transcriptome analysis of unfractionated peripheral blood leukocytes after exercise in human

**DOI:** 10.1038/s41598-023-38064-2

**Published:** 2023-07-10

**Authors:** Mingkun Nie, Qingling Liu, Ruoling Jia, Zhuoyi Li, Xiaoru Li, Xiangtao Meng

**Affiliations:** 1 School of Physical Education, Xinxiang University, Xinxiang, 453000 Henan China; 2 School of Pharmacy, Xinxiang University, Xinxiang, 453000 Henan China

**Keywords:** Computational biology and bioinformatics, Immunology

## Abstract

Exercise has profound but variable effects on the immune system. However, only limited information exists about the changes of exercise-induced gene expression in whole immune cells. The aim of this study is to unravel the potential molecular changes of genes which are related to immunity after exercise. The raw expression data and corresponding clinical of GSE18966 were downloaded from Gene Expression Omnibus database. The differentially expressed genes between control group and treat groups were performed by in-house developed perl scripts. A total of 83 differentially expressed genes (DEGs) (|log2 FC|> 1, FDR < 0.05) were identified between control and treat group 1 (0 h after exercise), 128 DEGs (|log2 FC|> 1, FDR < 0.05) between control and treat group 2 (4 h after exercise), and there was no significant difference between control and treat group 3 (20 h after exercise). Next, we identified 51 overlapping genes between treat group 1 (0 h after exercise) and treat group 2 (4 h after exercise) using Venn analysis. Protein–protein interaction (PPI) network was constructed by Cytoscape 3.7.2, and nine hub genes (S100A12, FCGR3B, FPR1, VNN2, AQP9, MMP9, OSM, NCF4, HP) were identified. Finally, 9 hub genes were identified as the potential biomarkers of exercise using validation set (GSE83578) verification analysis. These hub genes might serve as potential molecular targets of monitoring exercise and training processes in the further.

## Introduction

Exercise is known to offer a lot of healthy benefits, such as reducing the risks of cardiovascular disease, dementia, cancer, diabetes, depression, anxiety and obesity^1–5^. Thus, a wide range of different organizational bodies including the World Health Organization, the United Nations and many governments have been devoted to promoting physical exercise.

Numerous studies have been demonstrated that exercise has profounded but variable effects on the immune system^6–9^. It was found to be immune-protective in a number of studies, such as enhancement of wound healing and immune response^10–12^. In parallel, exercise has been demonstrated anti-inflammatory and immunomodulatory effects^13,14^. The regulation of immune is complex and which involves a complicated interaction of multiple immune cells, various cytokines, and chemokines. In order to understand more effective use of physical exercise in health promotion and disease, a thorough understanding of immunomodulatory effects needs to be acquired. However, that is currently lacking.

More recently, microarray has been used to monitor the peripheral blood leukocytes through the following exercise^15–19^. Noteworthy, nearly all studies reviewed were conducted with human peripheral blood mononuclear cells (PBMC) which comprised only a fraction of whole blood. Therefore, it is imperative to promote extensive research activities on the whole immune cells to provide more important information about exercise. Here, we downloaded the whole blood expression profiles after a bout of exercise from Gene Expression Omnibus (GEO) database. 83 differentially expressed genes were obtained between control and treat group 1 (0 h after exercise). While 128 DEGs between control and treat group2 (4 h after exercise). Then we identified 51 overlapping genes by Venn analysis. Furthermore, we constructed a PPI network of these genes by using the STRING database and screened out nine hub genes. Gene Ontology (GO) and Kyoto Encyclopedia of Genes and Genomes (KEGG) biological process enrichment of hub genes was performed.Finally, the nine hub genes were validated which used GSE83578 data set.The aim of this study might to gain more insight into the mechanism underlying the altered immunity, and unravel the potential molecular changes of genes related to immunity after exercise.

## Results

### Identification of DEGs

**Figure 1:**
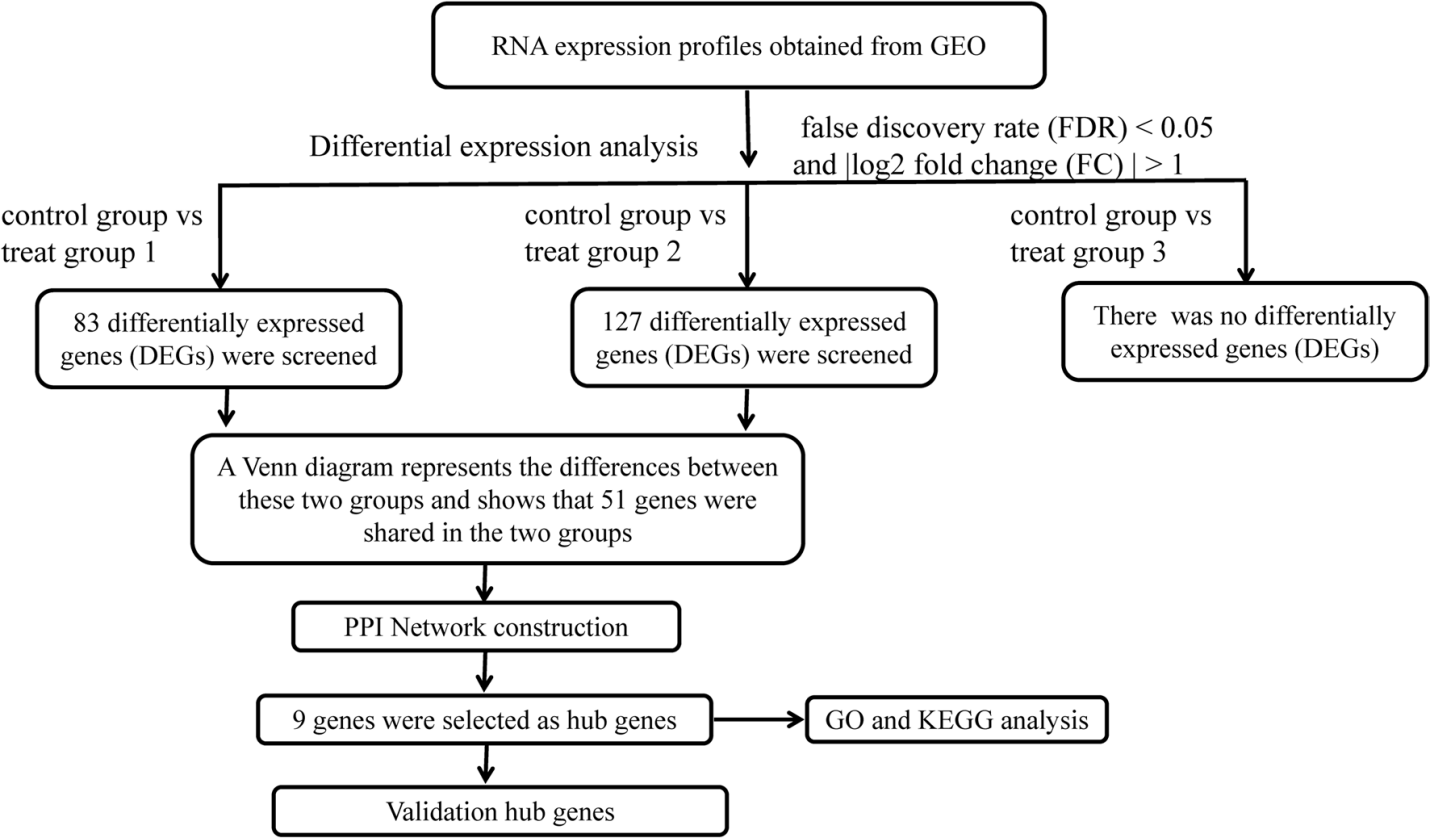
The flowchart describing the design of the study.

**Figure 2:**
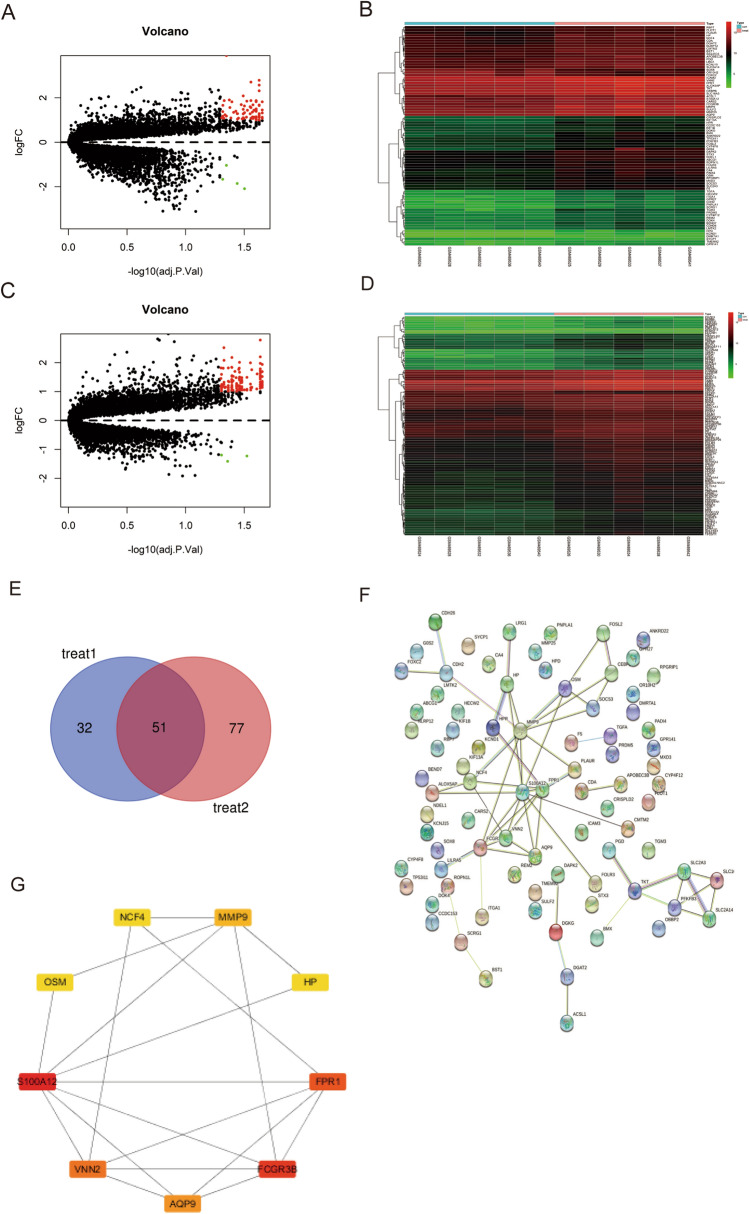
Differentially expressed genes (DEGs). (**A**) Volcano map shows differentially expressed genes between control group (pre-exercise) and treat group 1 (0 h post-exercise). Red dots represent significantly up-regulated DEGs, green dots represent significantly down-regulated DEGs and black dots represent non-significant genes. (**B**) Heatmap shows the expression levels of DEGs between control group (pre-exercise) and treat group 1 (0 h post-exercise), which was drawn in R (version 4.2.2) package ‘pheatmap’, (https://cran.rstudio.com/web/packages/pheatmap/index.html). Red represents highly-expressed genes and green represents low gene expression. (**C**) Volcano map shows differentially expressed genes between control group(pre-exercise) and treat group 2 (4 h post-exercise). (**D**) Heatmap shows the expression levels of DEGs between control group (pre-exercise) and treat group 2 (4 h post-exercise). (**E**) Venn diagram depicting the number of overlapping DEGs between treat group 1 and treat group 2. (**F**) The network of protein–protein interactions (PPI) of overlapping genes. (**G**) Top 10 hub genes of overlapping genes.

The flow chart of the analysis procedure is shown in Fig. 1. To determine the difference of gene expression between treat and control groups in unfractionated peripheral blood at the four different time-points (before and 0, 4 and 20 h after exercise), we conducted a differential analysis among control and treat groups with |log2 FC|> 1 and the cut off criteria of FDR < 0.05. A total of 83 DEGs were identified between control and treat group 1 (0 h after exercise). The volcano map (Fig. 2A) and heatmap (Fig. 2B) indicated that 79 DEGs were significantly up-regulated, while 4DEGs were down-regulated. There were 128 DEGs between control and treat group 2 (4 h after exercise) which included 3 significantly down-regulated genes and 125 up-regulated genes. The result was plotted by using a volcano plot (Fig. 2C) and heatmap (Fig. 2D). No changes in gene expression was significantly different between control and treat group 3 (20 h after exercise).The results could be caused due to variations in the stress effects at the organism level. The stress effects at the organism level could be enhanced by exercise which might persist after exercise for several hours, and the levels return to baseline after 24 h.

Then, Venn analysis was performed to identify the differentially expressed genes in common between treat group 1 (0 h after exercise) and treat group 2 (4 h after exercise), and the results showed that there were 51 overlapping genes (Fig. 2E).

### PPI network construction and screening of hub genes

To reveal the potential connection between overlapping genes, a PPI network of these genes was constructed using the STRING database. Removing the free nodes, the PPI network contained 56 nodes and 43 edges (Fig. 2F). Subsequently, 12 algorithms (MCC, DMNC, MNC, Degree, EPC, BottleNeck, EcCentricity, Closeness, Radiality, Betweenness, Stress, ClusteringCoefficient) were utilized for identification of hub genes and visualized by Cytoscape 3.7.2. A subnetwork with 5 nodes and 10 edges was mapped (Fig. 2G). This indicated that nine hub genes (S100A12, FCGR3B, FPR1, VNN2, AQP9, MMP9, OSM, NCF4, HP) might play an important role in immune function.

### Functional enrichment analysis of hub genes

**Figure 3:**
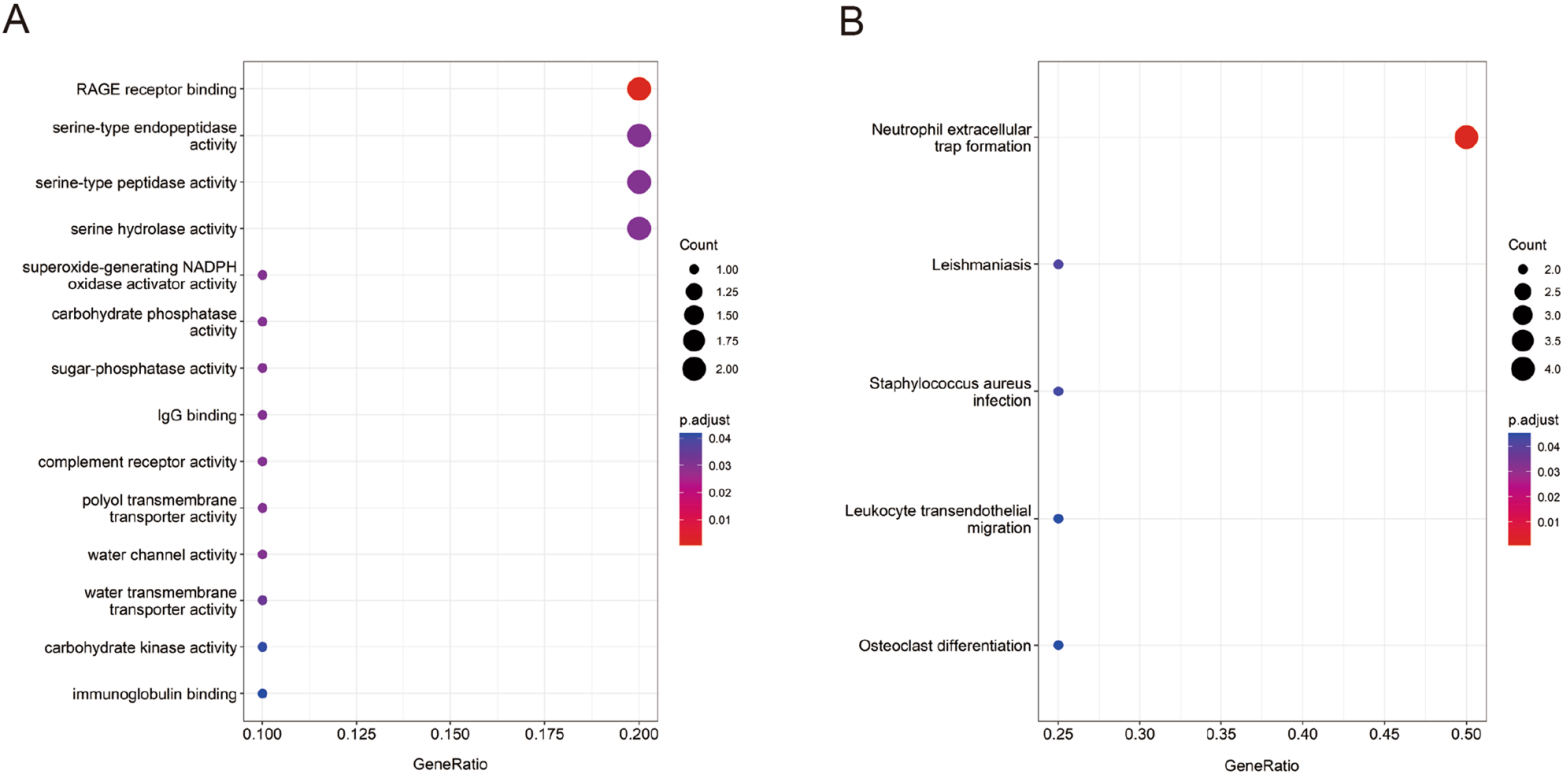
GO and KEGG enrichment analysis of hub genes. (**A**) Hub genes GO enrichment analysis. (**B**) Hub genes KEGG enrichment analysis. Images are obtained from KEGG (http://www.kegg.jp/kegg/kegg1.html) with permission.

To further investigate the potential functional of the hub genes, GO and KEGG enrichment analysis were carried out. The results of GO enrichment analysis revealed that these hub genes were mainly enriched in RAGE receptor binding, serine-type endopeptidase activity, serine-type peptidase activity, serine hydrolase activity, superoxide-generating NADPH oxidase activator activity, carbohydrate phosphatase activity, sugar-phosphatase activity, IgG binding, complement receptor activity, polyol transmembrane transporter activity, water channel activity, water transmembrane transporter activity, carbohydrate kinase activity, and immunoglobulin binding (Fig. 3A). Moreover, KEGG pathway enrichment analysis indicated that the 9 hub genes were mainly enriched in Neutrophil extracellular trap formation, Leishmaniasis, Staphylococcus aureus infection, Leukocyte transendothelial migration, and Osteoclast differentiation (Fig. 3B).

### Validation hub genes

**Figure 4:**
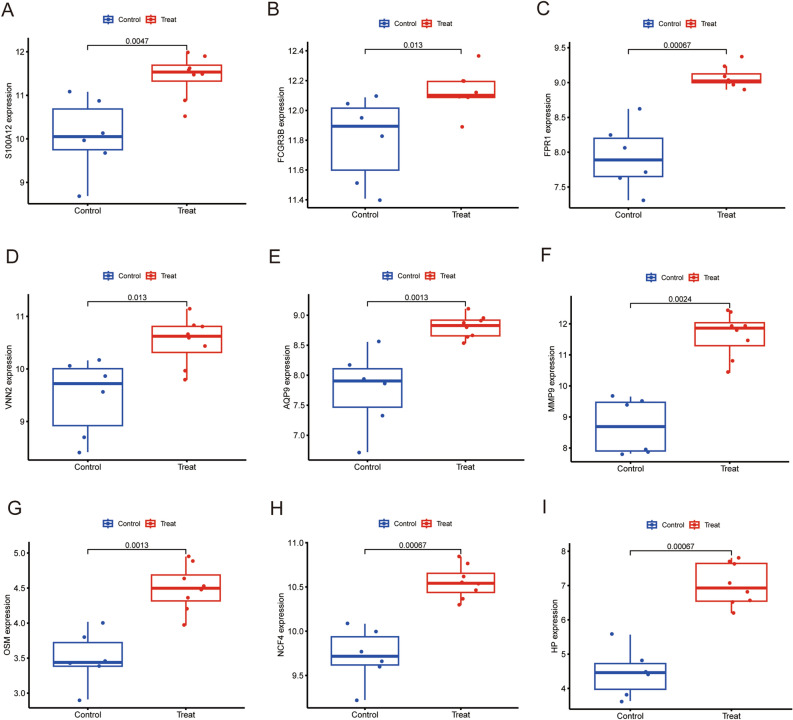
Boxplot of the 9 hub genes between before and 30 min after exercise in validation dataset (**A–I**). *P*-values < 0.05 were considered statistically significant.

**Figure 5:**
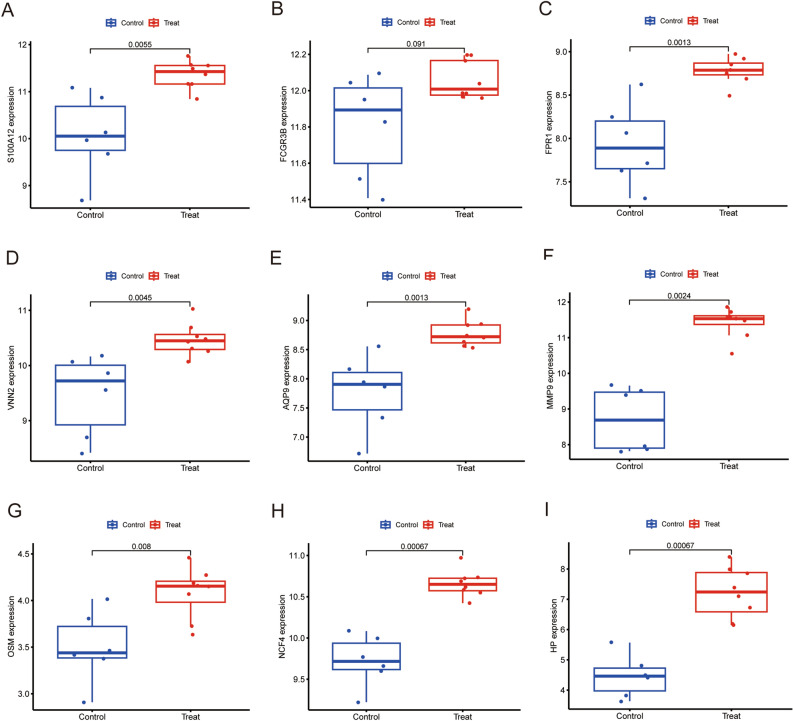
Boxplot of the 9 hub genes between before and 3 h after exercise in validation dataset (**A–I**). *P*-values < 0.05 were considered statistically significant.

**Figure 6:**
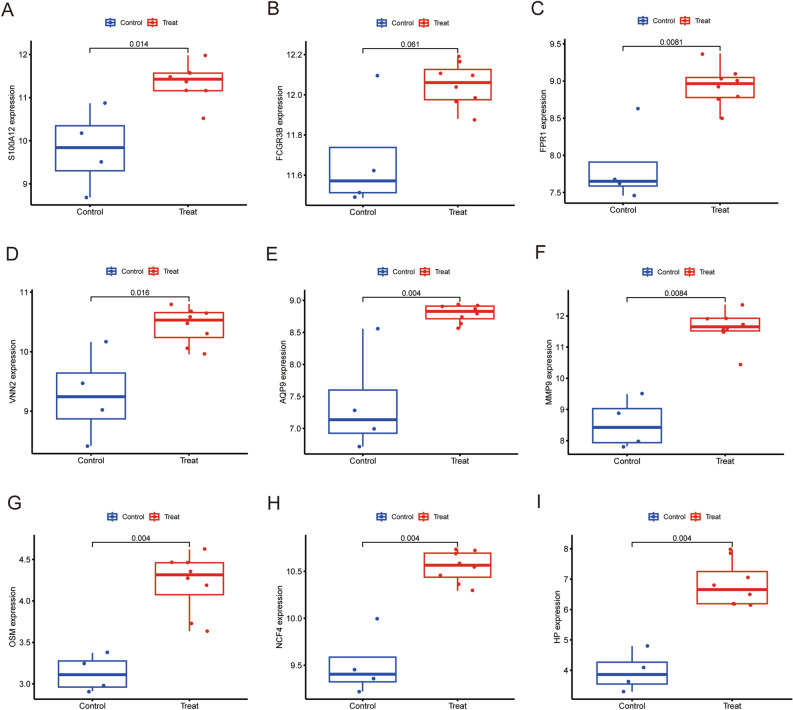
Boxplot of the 9 hub genes between before and after exercise in male athletes (**A–I**). *P*-values < 0.05 were considered statistically significant.

**Figure 7:**
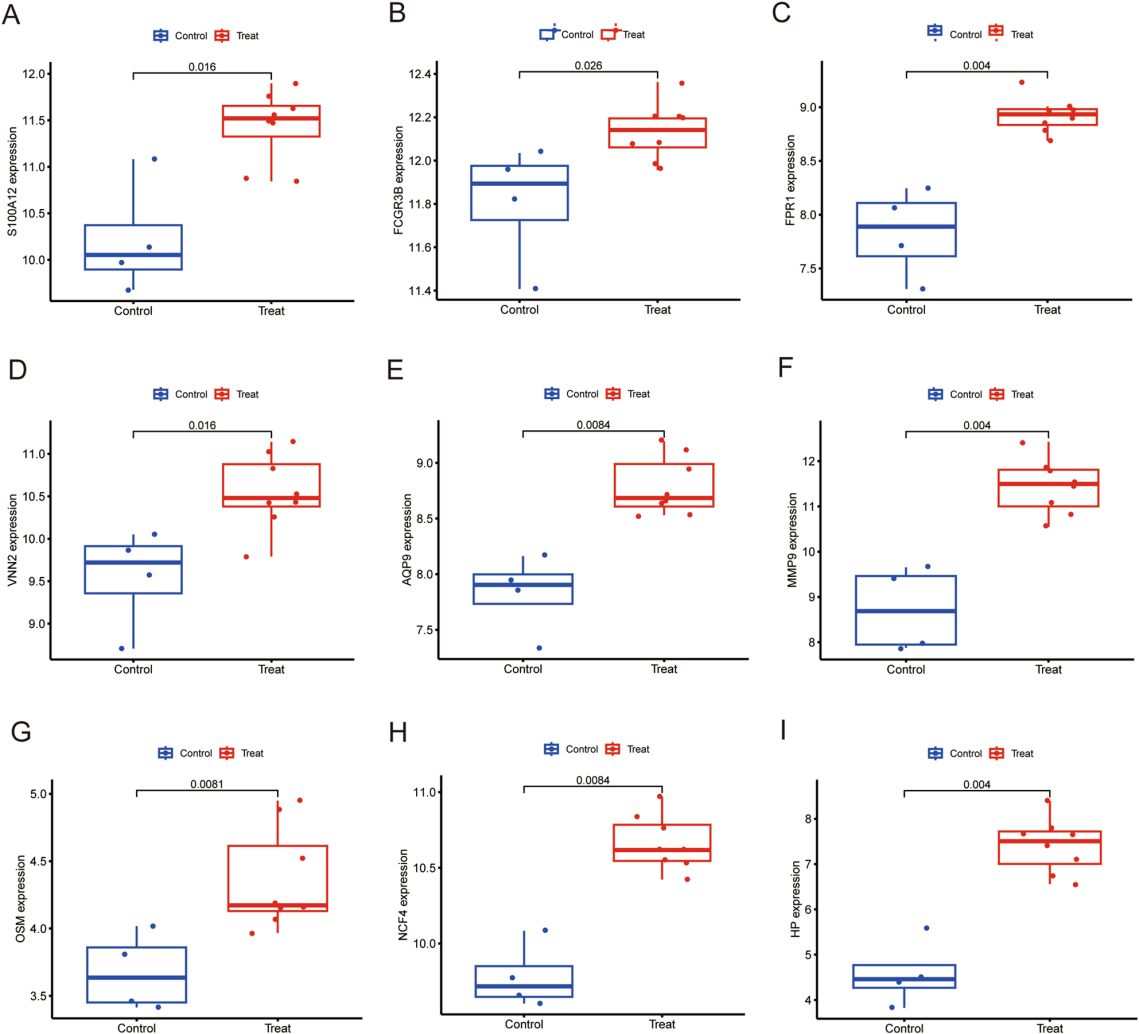
Boxplot of the 9 hub genes between before and after exercise in female athletes (**A–I**). *P*-values <0.05 were considered statistically significant.

To verify hub gene expression after exercise, we downloaded another online dataset GSE83578. We further confirmed the relationship between the 9 hub genes and the exercise, expression levels of these hub genes were detected to using Boxplot. Results of the analysis showed that all hub genes (S100A12, FCGR3B, FPR1, VNN2, AQP9, MMP9, OSM, NCF4, HP) were significantly up-regulated in 30 min (Fig. 4A–I) and 3 h (Fig. 5A–I) after exercise, but there were no changes in 24 h after exercise (Supplementary Figure S1A–I). The results are consistent with the training set. We also assessed the association between hub genes and sex of the athletes. The 8 hub genes (S100A12, FPR1, VNN2, AQP9, MMP9, OSM, NCF4, HP)were significantly up-regulated in male athletes between before and after exercise (Fig. 6A–I). The expression level of FCGR3B was not significant. Consequently, this needs to be explored further in future. The expression level of these 9 hub genes were significantly up-regulated in female athletes (Fig. 7A–I). In summary, S100A12, FCGR3B, FPR1, VNN2, AQP9, MMP9, OSM, NCF4 and HP could represent important biomarkers of immune for exercise.

### Receiver operating characteristic curves of verified hub genes

**Figure 8:**
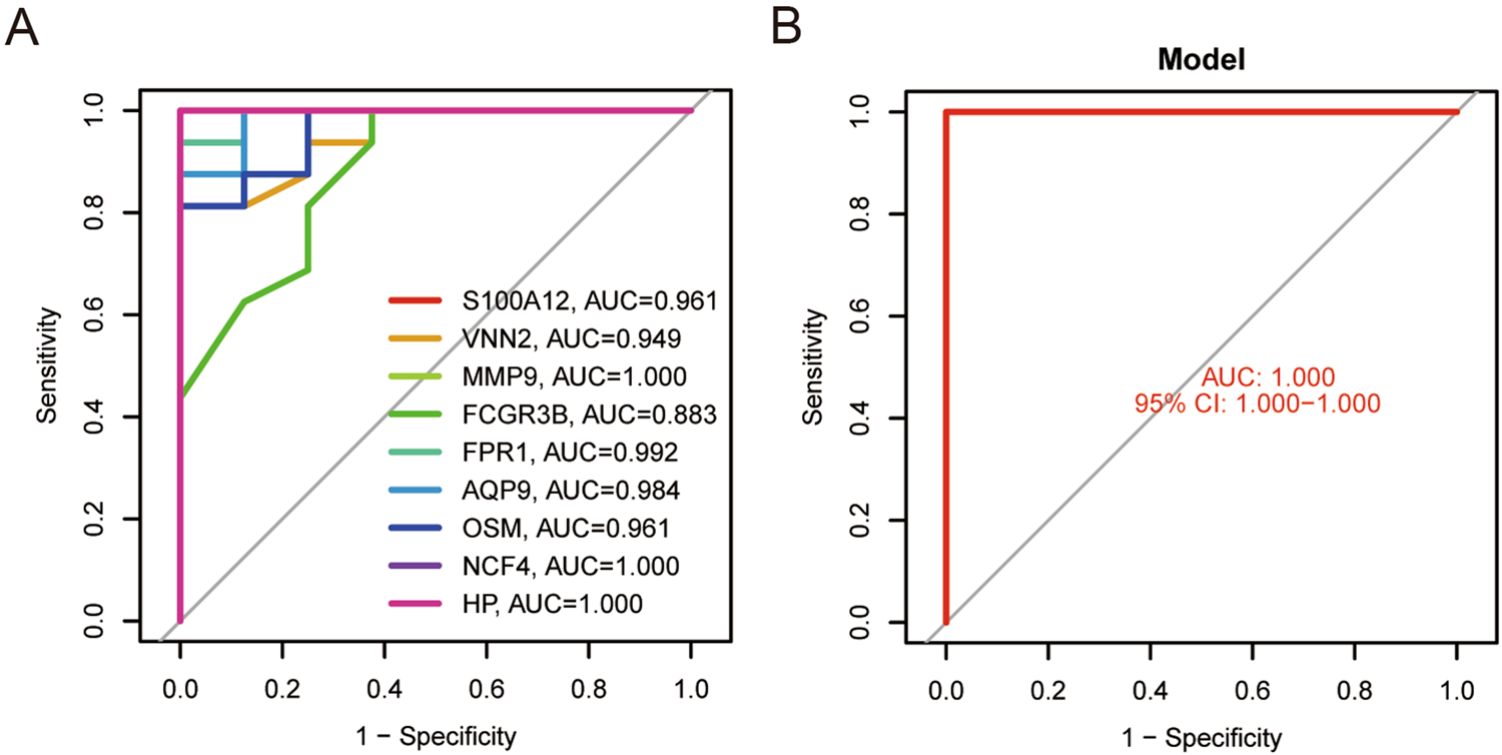
ROC curves of the 9 hub genes. (**A**) ROC curves of the 9 hub genes. (**B**) ROC curve of the combination model of the 9 hub genes.

We further investigated the diagnostic effectiveness of the 9 hub genes (S100A12, FCGR3B, FPR1, VNN2, AQP9, MMP9, OSM, NCF4, HP) using ROC curves. All 9 hub genes had a certain diagnostic accuracy with AUC values of over 0.9(Fig. 8A). The ROC analyses of 9 hub genes were performed. The AUC (95% CI) were 1.000 for all hub genes (Fig. 8B). Which indicated that the 9 hub genes might serve as potential molecular targets of monitoring exercise and training processes in the further.

## Discussion

Exercise has been shown to cause various physiological and biochemical changes within human body, such as oxidative stress, production of several cytokines including interleukins, inhibition or promotion inflammatory processes^20–23^. The relationship between exercise and immunity remains that the research should be focus on sport science^24,25^. Although several studies have reported that the microarray analysis was used to investigate the changes in gene expression due to exercise-induced, these analyses were largely limited to differences in peripheral blood mononuclearcells (PBMC)^26,27^.

In this study, we employed gene expression profiles of whole peripheral white blood cell RNA to monitor the changes in gene expression after exercise. First, differential expression analysis was performed in pre-exercise as compared with post-exercise. There were 83 DEGs between pre-exercise and post-exercise (0 h after exercise). A total of 128 DEGs were identified between pre-exercise and post-exercise (4 h after exercise), and no changes were significantly different between pre-exercise and post-exercise (20 h after exercise). Altogether, these findings suggested the level of mRNA expression peaked after four hours of exercise and remained baseline within 20 h after exercise.

Exercise is a complex, unique, controllable, quantifiable and repeated stress stimulation^28^. It can change body function, and also act as a key regulator of modulatory effects on immune function^29^.Increasing evidences shows that the exercise stress evokes inflammatory-like responses of the immune system with the activation of both proinflammatory and anti-inflammatory pathways^30^. The balance of both is mainly dependent on exercise type, intensity and duration. Therefore, moderate exercise has been shown to have an anti-inflflammatory effect with improved anti-infectious capabilities, while exhaustive exercise may temporarily reduce the individuals’ immune competence, enhance stimulate. In this study, we mined the hub genes from moderate exercise and validated in exhaustive exercise. In both of these exercise, the expression of nine hub genes were increased before and the first few hours after exercise. These findings showed exercise could enhance variation of gene expression. However, in both of these exercise, the expression of nine hub genes were returned to baseline 24 h after exercise. This variation might be due to the following reason. The systemic inflammatory response to the bout of exercise was characterized by promoting the expression of some immune-related genes or the secretion of immune factors^31,32^. 24 h immune-related gene shad returned to baseline concentrations which suggested the resolution of the systemic inflammatory response.

To gain further regarding changes in whole peripheral white blood transcriptome after exercise, the present analysis focused on changes in whole peripheral white blood transcription between 0 and 4 h after exercise. Using Venn analysis, a total of 51 overlapping genes were identified between the two groups. Then, nine hub genes (S100A12, FCGR3B, FPR1, VNN2, AQP9, MMP9, OSM, NCF4, HP) was screened out using Cytoscape 3.7.2. These six hub genes have previously been described in a variety of immune responses. The changes of OSM and NCF4 were also obtained from the original studies^9^. Manoj Khokhar et al. conformed that the expression levels of VNN2 were up-regulated in both yoga and exercise^33,34^. Rullman et al. found that the plasma levels of MMP-9 were increased at both 27 and 57 min of exercise^35^. While MMP9 up-regulated in older (≥ 65 years) endurance-strength training^36^. These results were in agreement with our results. Of note, data of FCGR3B expression showed that the level of FCGR3B were higher expression in the NK cell-mediatedcytotoxicity pathway following exercise in the early-pubertal girls^37^. Moreover, the expression of FCGR3B was up-regulated in older (≥ 65 years) endurance-strength training^36^. In contrast, some studies also indicated that the level of FCGR3B in the trained muscle were lower expression than other exercised or non-exercised untrained muscle^38,39^. These finding suggested that the dual effects of FCGR3B in exercise needed further explored. A wide array of studies documented that S100A12 plays an important role in immune regularly, antimicrobial functions, induction of signal transduction and cell proliferation^40–43^. Interestingly, Hossein Khorramdelazad et al. found that S100A12 serum levels were significantly elevated at 24 h post exercise^44^. This result went against our findings. One of the most important reasons for the opposite conclusion was that S100A12 in different form of exercise could exert different physiological effects.

Remarkably, our analyses using the same dataset as that in Pu and Sun^45^. As found in L. Pu & P. Sun, 433 up-regulated DEGs and 286 down-regulated DEGs were identified between pre-exercise and 0 h post-exercise. Subsequently, 3 up-regulated hub genes (VEGFA, POMC, and NRAS) and 3 down-regulated hubgenes (HRAS, NCOR1, and CAV1) were screened out using PPI network. These results were different from this research finding. There could be several reasons for this difference. First, the inconsistency in results might be due to the use of different analytical tools. Because of different algorithms of each tool, the results could vary. Second, as for the differential expression analysis differed in various time periods after exercise, some variance might be expected between these two studies.

Yet, this study has a number of limitations. Firstly, we just derived the research data from the GEO public databases. Secondly, the function of the 9hub genes have not been verified with biological experiments. Thirdly, data on all participants were collected from a single dataset and the number was relatively small.

## Conclusions

Through comparing the changes between 0 h after exercise and 4 h after exercise based on whole peripheral white blood transcription, we mined nine hub genes which might be criticalto the alteration of immunological state and immune function in human. These hub genes might serve as potential molecular targets of monitoring exercise and training processes in the further.

## Methods

### Data collection

The gene expression profiles of a bout of exercise were downloaded from the Gene Expression Omnibus GSE18966 dataset (PMID: 19945435)^9^ (https://www.ncbi.nlm.nih.gov/geo/), which contained the expression data of peripheral blood from 5 healthy consenting volunteers (male, age 44.2 ± 9.4 years) before and 0, 4 and 20 h after exercise. As described in the original study, all participants were healthy and physically active, but were not currently engaged in any kind of resistance or endurance exercises. All participants were asked not to eat food and drink alcohol, coffee, or tea for 12 h before the exercise and were asked to report in the laboratory at 8:30 am. Then, they started exercise on an electrically braked cycle ergometer at 80% of their predicted maximum workload for 4 h with rest at every 1 h. Blood was collected by venouspuncture at 4, 8 and 24 h after the start of exercise.

### Identification of differentially expressed genes

This dataset were studied during four time periods: before exercise (control group), 0 h after exercise (treat group 1), 4 h after exercise (treat group 2) and 20 h after exercise (treat group 3). Differentially expressed genes (DEGs) were obtained between control group and treat groups using in-house developed perl scripts with the cut-off criteria of false discovery rate (FDR) < 0.05 and |log2 fold change (FC) |> 1. Moreover, volcano plots were generated with the R limma package, and heat maps was conducted by the R “pheatmap” package. Subsequently, we carried out a Venn diagrams to analyze the specific overlap in genes among groups from using the InteractiVenn website (http://bioinformatics.psb.ugent.be/webtools/Venn/).

### PPI network construction and hub genes selection

An analysis of overlapping genes-protein interaction networks was performed based on the STRING protein interaction database (http://string-db.org/), and 0.4 was defined as the threshold for interaction score. Then, Hub genes were screened out by considering the high degree of connectivity in the PPI networks using the cytohubba plugin of Cytoscape 3.7.2.

### Functional enrichment analysis

Gene Ontology (GO) and Kyoto Encyclopedia of Genes and Genomes (KEGG) biological process enrichment of hub genes was performed by R statistical software including packages of “clusterProfiler”, “org.Hs.eg.db”, “enrichplot”, “ggplot2”, and “GOplot”. An adjusted *p*-value < 0.05 was regarded as statistically significant.

### Validation hub genes

To validate the hub genes, we downloaded the gene expression profiles of exhaustive exercise (half-marathon) from the Gene Expression Omnibus GSE83578 dataset (PMID:27832807)^46^ (https://www.ncbi.nlm.nih.gov/geo/), which contained the expression data of peripheral blood from 8 well-trained male athletes [34.8 ± 9.4 years, body mass index (BMI) 23.41 ± 2.2 kg/m^2^] and 8 well trained female athletes [38.5 ± 5.7 years, body mass index(BMI) 21.9 ± 1 kg/m^2^]. All athletes had to be nonsmokers and none suffered from acute or chronic diseases or reported intake of medication or antioxidant. The blood samples were taken pre-exercise and post-exercise (30 min, 3 h, 24 h). For defining the differential expression of the hub genes, Boxplot were draw in R using the ggplot and ggpubr packages. A cutoff *p*-value of 0.05 was used as the differential cutoff. We further performed ROC and calculated AUC and C-index to validate these hub genes.

## Supplementary Information

Supplementary Information 1.

Supplementary Information 2.

## Data Availability

The datasets generated and/or analysed during the current study are available in the Gene Expression Omnibus (GEO) database (https://www.ncbi.nlm.nih.gov/geo/) with Accession Numbers: GSE18966 and GSE83578.
