# Supplementary material for: Comparative transcriptome analysis of unfractionated peripheral blood leukocytes after exercise in human
Source: Sci Rep. 2023 Jul 10;13:11140. doi: 10.1038/s41598-023-38064-2 (PMC10333281; doi:10.1038/s41598-023-38064-2)
Supplement: Supplementary file 2 — Supplementary Information 2. [file 41598_2023_38064_MOESM2_ESM.docx]

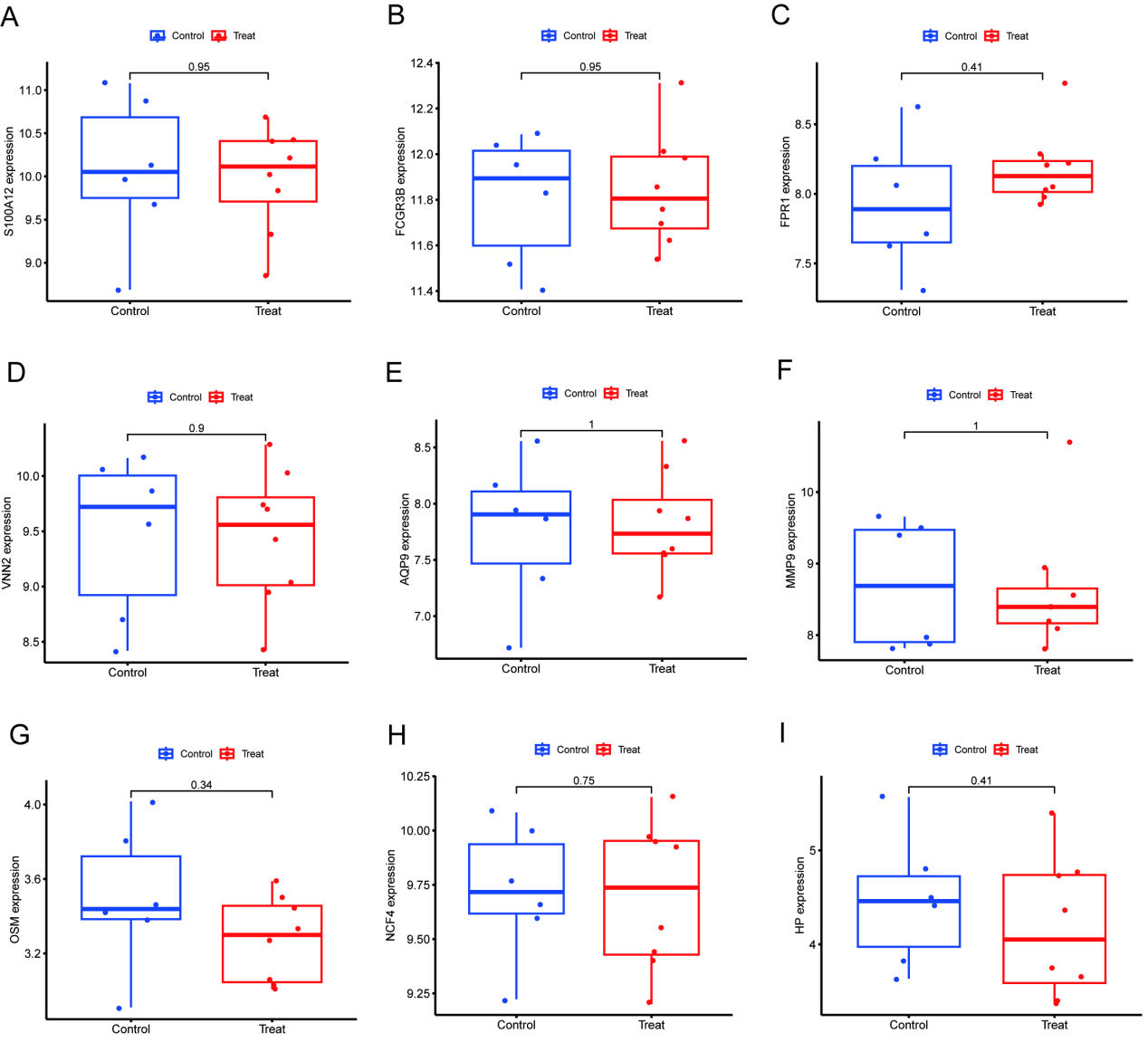


Supplementary Figure. S1 Boxplot of the 9 hub genes between before and 24 hours after exercise invalidation dataset(A-l). P-values less than 0.05 were considered statistically significant.
